# GS-YOLO: A lightweight high-accuracy model for small target detection in drone aerial images

**DOI:** 10.1371/journal.pone.0350840

**Published:** 2026-06-08

**Authors:** Xiaoyuan Jin, Xiyuan Zhu, Dongdong Kang, Wangyu Shen, Yang Zhao, Xun Li, Baoxi Yuan, Yuzhen Zhao

**Affiliations:** 1 Shaanxi Key Laboratory of Liquid Crystal Polymer Intelligent Display, Technological Institute of Materials & Energy Science (TIMES), Xijing University, Xi’an, China; 2 School of Electronic Information, Xijing University, Xi’an, China; Communication University of Zhejiang, CHINA

## Abstract

For the problems of weak feature representation, significant scale variation, and background interference in small target features of unmanned aerial vehicle (UAV) aerial images, existing detection methods struggle to achieve both lightweight deployment and detection accuracy. Therefore, this paper proposes an extremely lightweight and accurate small target detection architecture named GS-YOLO. Through modular innovation, it achieves extreme lightness and improved detection performance. The core innovations include: 1) Design of a lightweight small target perception attention fusion module C2FGhostLight, using proportionally optimized GhostConv to replace traditional convolution, combined with a dual-path lightweight attention mechanism, which significantly reduces parameters while dynamically suppressing background interference; 2) Proposal of a lightweight channel attention module for small target perception (SOLCA), through a “channel focusing-local enhancement” dual-branch compact structure and adaptive weighted fusion, to strengthen weak feature representation. Experimental results show that on the VisDrone public dataset, GS-YOLO improves mAP50 by 0.9% compared to YOLOv8n, with a model parameter size of only 0.84M. It maintains lightweight characteristics and provides a solution for engineering applications in UAV aerial photography scenarios.

## 1. Introduction

In recent years, the rapid development of computer vision technology has expanded intelligent applications across diverse domains. Drones, endowed with advantages such as flexible deployment, broad coverage, and all-weather operational capability, have emerged as the primary platform for deploying these technologies. Consequently, drone target detection technology has emerged, offering widespread utility in daily applications and serving as a critical enabler for intelligent systems. Its efficacy has been validated in precision agriculture [1], security surveillance [2–3], disaster response [4], infrastructure inspection [5], and numerous other fields [6–9].

However, drones’ high flexibility in flight altitude and imaging angles results in significant scale variations among targets in captured imagery. Target sizes exhibit extreme disparity, spanning from tens to thousands of pixels. Such drastic scale differences impose stringent demands on the generalization capabilities of detection networks.

The research on UAV target detection has primarily evolved along two technical pathways: traditional machine learning and deep learning approaches. Traditional methods, reliant on manually designed feature extractors, exhibit notable limitations in UAV applications. On one hand, the UAV’s overhead perspective induces complex and variable target postures, rendering manual features inadequate for capturing nonlinear appearance deformations and cross-scenario generalization. On the other hand, these methods heavily depend on large-scale labeled datasets, yet UAV images often contain densely packed small targets and chaotic backgrounds, necessitating extensive manual annotation to construct such datasets — a process that hinders efficient dataset creation. In recent years, deep learning-based detection algorithms have become dominant, categorized into two-stage and single-stage methods. Two-stage approaches (e.g., R-CNN [10], Faster R-CNN [11], Mask R-CNN [12]) achieve high accuracy but suffer from redundant computations, compromising inference efficiency. Single-stage methods (e.g., Centernet [13], SSD [14], RetinaNet [15], YOLO [16–18]) bypass candidate region generation, directly extracting features while simultaneously performing classification and localization. This streamlined process ensures faster detection speeds without sacrificing accuracy, making single-stage methods more suitable for UAV target detection requirements.

The “You Only Look Once” (YOLO) [19] series has garnered significant attention due to its exceptional performance and broad application across detection tasks. However, optimizing YOLO models for precise identification of small targets in aerial imagery remains a critical challenge in current research. Small targets exhibit weak feature representation and are prone to background interference, complicating detection efforts. In long-distance aerial monitoring scenarios, detection algorithms must possess robust feature extraction capabilities and multi-scale perception. Current mainstream single-stage detectors primarily depend on convolutional neural network (CNN) architectures, whose localized receptive field limitations hinder full capture of distant target correlations and adaptation to scale variations, occlusions, and blurred boundaries in drone imagery. While Transformer-based methods [20–21] reduce errors in dense target detection and enhance efficiency in such scenarios, they increase model parameters and still struggle with small target recognition. Further improvements to these approaches are necessary.

Existing methods struggle to efficiently optimize the information fusion process and fail to fully leverage small target feature information, increasing the likelihood of missed or false detections during operations. To address the challenge of small target detection in complex scenarios, this paper proposes the GS-YOLO model. The main contributions of this paper are as follows:

1. Addressing the challenges of weak feature representation, significant scale variation, and severe background interference in unmanned aerial vehicle (UAV) small target detection, this paper proposes the GS-YOLO lightweight high-precision detection framework to effectively address small target detection in complex UAV imagery scenarios.
2. Designing a lightweight small target perception attention fusion module C2FGhostLight, this module employs proportionally optimized GhostConv for lightweight feature extraction, integrates a dual-path lightweight attention mechanism to dynamically suppress background noise, and balances computational efficiency while enhancing small target feature representation.
3. Proposing a lightweight channel attention module for small target perception (SOLCA), this module utilizes a compact dual-branch parallel structure to simultaneously achieve channel-level feature focusing and localized detail enhancement for small targets. It dynamically adjusts branch contributions via adaptive weighted fusion, markedly improving the model’s sensitivity to small targets under lightweight constraints.

The structure of this paper is as follows: Chapter 2 reviews and summarizes the latest research progress in UAV target detection. Chapter 3 focuses on the improved model designed for UAV image small target detection. Chapter 4 and Chapter 5 introduce the experimental environment configuration and parameter settings, and analyze and verify the results of multiple experiments conducted on the VisDrone2019 [22] dataset and UAVDT dataset. Chapter 6 summarizes the research work of this paper and discusses the potential directions for future related research.

## 2. Related work

### 2.1. Problem of weak characteristics of minor goals

Compared to ordinary images, drone imagery exhibits a distinct characteristic where targets are generally smaller due to the platform’s aerial nature. Repeated down-sampling reduces resolution, causing small target information loss and hindering effective feature extraction via traditional methods. Existing solutions often rely on high-resolution inputs to improve detection, but this increases computational and memory demands, restricting deployment on resource-limited devices.

Addressing small target detection challenges, prior research has proposed diverse approaches with inherent limitations. Guo et al. [23] introduced a dense connection mechanism via the C3D module to replace YOLOv8’s C2f module, though computational costs surged. Wang et al. [24] enhanced global and contextual feature capture by integrating small target detection structures into YOLOv8’s Neck module, but this increased parameter counts. Zhu [25] adopted transformer prediction heads to improve small-scale object recognition, yet model complexity and parameter size remained prohibitive for constrained platforms. Lan et al. [26] focused on convolution features via learned pixel displacement but neglected global context, limiting small target accuracy. Qin et al. [27] utilized high-resolution feature maps to enhance small target regions, achieving a 2.9% AP improvement on VisDrone2019, but network complexity and high-resolution processing inefficiencies persisted.

To resolve these issues, this paper introduces the GS-YOLO lightweight architecture, integrating the C2FGhostLight and SOLCA modules for collaborative small target optimization. C2FGhostLight replaces traditional convolution with proportionally optimized GhostConv, reducing 60% of feature extraction overhead while enhancing weak features via a specialized branch. SOLCA employs a “channel focusing-local enhancement” dual-branch structure to refine small target details, achieving both lightweight design and robust feature representation. This dual-module approach balances feature retention and computational efficiency, overcoming limitations of prior methods.

### 2.2. Background interference issues

The unmanned aerial vehicle (UAV) leverages its agile flight capabilities to significantly boost data acquisition efficiency. However, in complex scenarios, small targets in UAV images often exhibit sparse visual features and low pixel proportions, rendering traditional detection methods inadequate for precise localization and identification. Especially in cluttered backgrounds, detectors are prone to interference from environmental noise. While existing research has attempted to address these issues, notable limitations persist.

Lu et al. [28] introduced a hybrid patch embedding component to extract low-level features like edges and corners, aiding adaptation to complex backgrounds. However, this method prioritizes low-level feature extraction over high-level semantic processing, limiting its effectiveness in handling occlusions and blurred edges for small target recognition. Zhang et al. [29] proposed a novel attention mechanism to guide the network toward key regions while avoiding high computational costs. Yet, this approach remains insensitive to fine-grained details and fails to fully resolve detection challenges in complex environments. Lu et al. [30] designed the pure convolution-based feature extraction module ConvSimCB, which improves convolutional features but neglects global context capture and background noise suppression. Tang et al. [31] utilized SimAM to suppress background noise and enhance target features in maritime scenes, though its effectiveness diminishes under severe interference, leading to misclassification of reflective areas as targets and increased false positives. Rermborirak et al. [32] combined Nile Red fluorescence staining, YOLOv8, and a yellow filter to reduce background interference. However, limited microscope resolution and impurity-related misclassification degraded overall accuracy.

To address background noise interference, this paper introduces the lightweight attention fusion module C2FGhostLight. This module embeds a dual-path lightweight attention mechanism: channel attention dynamically filters relevant small target feature channels while suppressing irrelevant background responses, and spatial attention aligns the receptive field with small targets via 3 × 3 convolutions to precisely localize targets and suppress noise. The synergistic dual-path attention achieves two-dimensional background suppression within a lightweight framework, preserving weak small target features while overcoming prior limitations such as incomplete background suppression and susceptibility to false detections.

### 2.3. Scale variability issues

The unmanned aerial vehicle (UAV) operates at variable flight altitudes and angles, causing significant variations in target distances and scale distributions in captured imagery. This presents stringent demands on detector robustness to handle multi-scale targets coexisting in complex backgrounds.

Current mainstream detection methods struggle to effectively adapt to such multi-scale scenarios. Lou et al. [33] proposed an innovative downsampling technique to improve small target detection in dense configurations, yet overall performance gains were limited. Li et al. [34] adopted Bi-PAN-FPN’s multi-scale fusion strategy and replaced convolutions with GhostBlockV2, enhancing accuracy but failing to outperform comparable models. Wang et al. [35] introduced the FFNB module using BiFormer attention, reducing missed detections but still underperforming on tiny targets. Xu et al. [36] incorporated attention mechanisms for feature extraction but struggled to fully exploit scale-specific features, leaving tiny target detection suboptimal.

To address these challenges, this paper proposes the SOLCA module, a dual-branch lightweight channel attention design for multi-scale adaptation. The channel attention branch focuses on global semantic features for medium/large targets, while the local enhancement branch uses 3 × 3 convolutions to extract fine-grained details for tiny targets. Adaptive fusion weights dynamically balance branch contributions based on target scale distributions, enabling precise multi-scale feature aggregation. Channel compression and non-group convolution designs ensure lightweight implementation without parameter expansion, resolving prior trade-offs between multi-scale adaptability and model efficiency.

## 3. Method

### 3.1. C2FGhostLight

**Fig 1:**
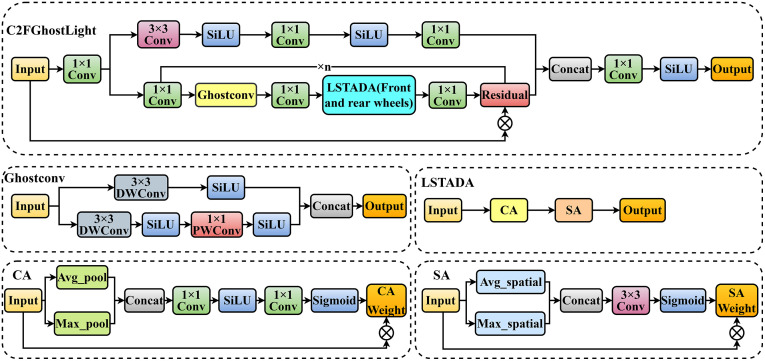
Structure of C2FGhostLight.

To address the core challenges in drone-based small target detection—including drastic scale variations, weak feature representation of small targets, chaotic background interference, and the difficulty in balancing lightweight design with feature integrity — this paper proposes the C2FGhostLight lightweight small target perception attention fusion module. The module employs proportionally optimized GhostConv layers for lightweight feature extraction, striking a balance between computational efficiency and feature retention. A small target-specific branch with receptive field alignment enhances weak feature representation, while an embedded dual-path lightweight attention mechanism highlights target regions and suppresses background noise. The module’s architecture is depicted in Fig 1.

For the input feature map, C2FGhostLight employs a four-stage sequential processing pipeline for feature enhancement and fusion. Initially, a 1 × 1 convolution compresses the input feature map. The intermediate channel count is determined by relaxing the output channel compression ratio to 0.6, preventing excessive loss of small target features. Subsequently, the feature map is divided into three parallel branches along the channel dimension: a global semantic branch, a local detail branch, and a small target-specific branch.

The dual-path lightweight attention is the core component for balancing lightweight module and feature integrity. The channel attention weights and spatial attention weights are calculated respectively as shown in formula (1) and (2):


$$\begin{array}{c} {\text{W}}_{\text{ca}}=\sigma \;\left({\text{W}}_{\text{2}}\cdot \text{SiLU }({\text{W}}_{\text{1}}\cdot \text{Cat}({\text{X}}_{\text{avg}},{\text{X}}_{\text{max}})+{\text{b}}_{\text{1}}+{\text{b}}^{\text{2}}\right) \end{array}$$ (1)

Among them,X_avg_ and X_max_ represent the results of global average pooling and maximum pooling of the input feature map, respectively. Cat(⋅) denotes the operation of concatenating channel dimensions; W_1_ and b_1_ are the weights and biases of the first convolutional layer, while W_2_ and b_2_ are the weights and biases of the second convolutional layer; σ is the Sigmoid activation function. This formula fuses the global context and local salient features through double pooling, and simultaneously uses dynamic channel compression to control the computational cost. Under the premise of lightweighting, it improves the filtering accuracy of channel attention for small target-related channels.


$$\begin{array}{c} {\text{W}}_{\text{sa}}=\sigma \;\left({\text{W}}_{\text{3}}\cdot \text{Cat}({\text{X}}_{\text{avg}\backslash \_\text{spa}},{\text{X}}_{\text{m}ax\backslash \_\text{spa}}+{\text{b}}_{\text{3}}\right) \end{array}$$ (2)

Among them, X_avg\_spa_ and X_max\_spa_ represent the average and maximum pooling results of the feature map after channel attention weighting in the spatial dimension; W_3_ and b_3_ are the weights and biases of the 3 × 3 convolution layer. The receptive field of the 3 × 3 convolution matches the typical size of small targets, avoiding the redundant computation of large kernel convolution and achieving precise spatial positioning under lightweight implementation.

After the features of each branch are weighted by attention, the final aggregation is achieved through the feature fusion weighting formula (3):


$$\begin{array}{c} {\text{F}}_{\text{out}}=\text{Con}{\text{v}}_{\text{1}\times \text{1}}\left(\sum_{\text{i}=\text{1}}^{\text{3}}\alpha_{\text{i}}\;{\text{F}}_{\text{fusion},\text{i}}\right)+\text{X}\times \text{0}.\text{8} \end{array}$$ (3)

Among them, F_fusion,1_, F_fusion,2_, and F_fusion,3_ represent the refined features of global semantics, local details, and small target-specific branches respectively; α_i_ = cos (F_fusion,i_, F_total_)/∑^3^_j=1_ cos (F_fusion,j_, F_total_) is the branch weight coefficient, which is used to dynamically allocate the contribution of each branch; Conv_1 × 1_(⋅) achieves channel adaptation, and 0.8 times residual attenuation is used to avoid the enhanced features being masked by the original residuals. The specific reason for using 0.8 is explained in Section 5.3. This formula allows the features of the small target branch to obtain more weight through similarity weighting, and replaces the direct concatenation of multiple branches with 1 × 1 convolution to reduce redundant computations, further balancing the feature integrity and lightweight requirements.

### 3.2. SOLCA

**Fig 2:**
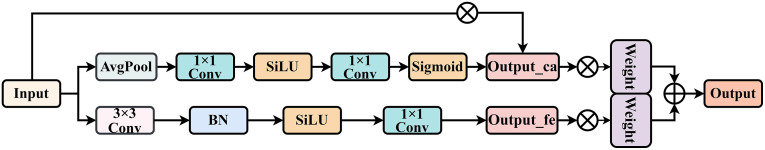
Structure of SOLCA.

To address core challenges in unmanned aerial vehicle small target detection — including weak feature representation, significant background interference, and the difficulty in balancing lightweight design with detection accuracy — this paper proposes the Small-Object-Aware Lightweight Channel Attention (SOLCA) module. This module employs a dual-branch parallel structure to concurrently achieve channel-level feature focusing for critical elements and localized detail enhancement for small targets. Adaptive weighted fusion dynamically balances branch contributions, controlling computational overhead while enhancing detection sensitivity. Its architecture is illustrated in Fig 2.

The SOLCA module’s core design prioritizes small target adaptation and lightweight optimization, comprising four key components. The lightweight channel attention branch employs a streamlined architecture consisting of global pooling, channel compression, activation, restoration, and weight generation steps, sequentially extracting channel-level global semantic information. After two layers of 1 × 1 convolutions, it finally generates the channel attention weight W_ca_ through the Sigmoid function. The formula is as follows:


$$\begin{array}{c} {\text{W}}_{\text{ca}}=\;\sigma \left(\overset{\text{2}}{\underset{\text{1}\times \text{1}}{\text{Conv}}}(\text{SiLU}\left(\overset{\text{1}}{\underset{\text{1}\times \text{1}}{\text{Conv}}}\left(\text{GlobalAvgPool}\left(\text{x})\right)\right)\right)\right) \end{array}$$ (4)

Among them, σ represents the Sigmoid activation function, Conv^1^_1 × 1_ and Conv^2^_1 × 1_ respectively denote the channel compression and reduction convolution layers, and x represents the input feature map.

The small target feature enhancement branch uses a 3 × 3 convolution as the core extraction unit. With padding = 1 and stride = 1, the feature map size remains unchanged to avoid the fragmentation of small target information caused by group convolution. After stable training with BatchNorm2d and activation by SiLU, it is restored to the input channel number c_1_ through a 1 × 1 convolution. Adaptive fusion weights introduce learnable parameters, and after Softmax normalization, the contribution degrees of the two branches are dynamically allocated. The fusion formula is:


$$\begin{array}{c} {\text{out}}_{\text{fusion}}=\omega_{1}\;\left({\text{W}}_{\text{ca}}⊙\text{x}\right)+\omega_{2}\;\text{fe}(\text{x}) \end{array}$$ (5)

Among them, ⊙ represents the element-wise multiplication operation, and fe(x) is the output feature of the small target feature enhancement branch.

The forward propagation process of the module is straightforward and efficient. The input feature map x is first processed by the ca branch to produce channel-enhanced features and by the fe branch to generate local fine-grained features fe(x). These two feature streams are then weighted, fused, and passed through an output convolution layer to produce the final enhanced features.

The SOLCA module’s core advantages stem from three key aspects: its dual-branch architecture collaboratively strengthens global semantic features and local detailed features, addressing the challenges of weak small target representation and background interference; lightweight strategies like channel compression and 1 × 1 convolutions replace computationally heavy large-kernel operations; and its highly compatible design allows seamless integration into YOLO series frameworks, enabling the GS-YOLO architecture to achieve both lightweight efficiency and robust performance.

### 3.3. GS-YOLO

**Fig 3:**
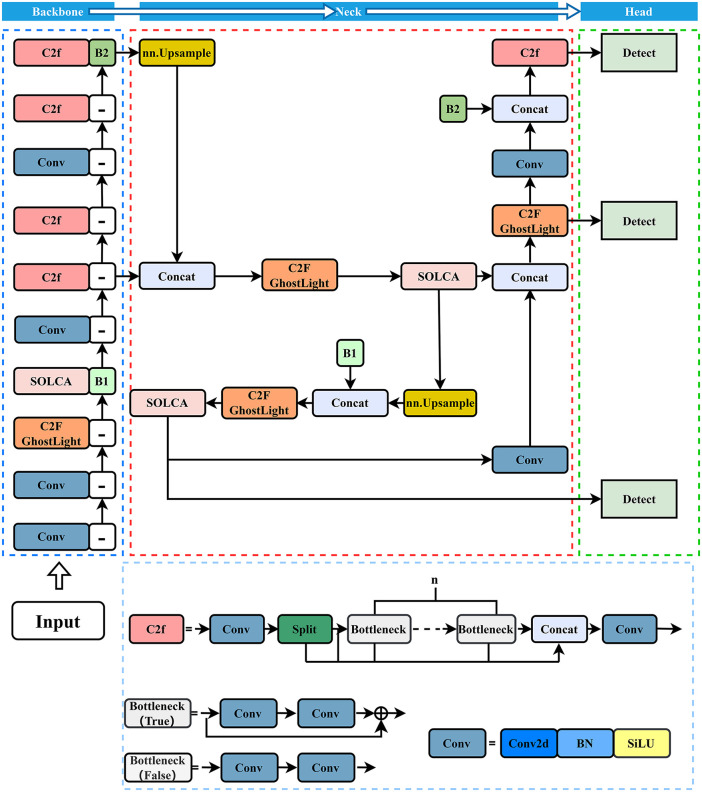
Structure of GS-YOLO.

To address core challenges in unmanned aerial vehicle small target detection — including weak target features, severe background interference, and drastic scale variations — this paper proposes an improved GS-YOLO detection network based on YOLOv8. The architecture integrates three core innovations: First, the C2FGhostLight module achieves lightweight feature extraction and background suppression through optimized GhostConv replacements and a dual-path attention mechanism, which highlights small targets while enhancing weak feature expression. Second, the SOLCA module employs a “channel focusing-local enhancement” dual-branch design, where the channel branch selects key features and the local branch amplifies fine-grained details; adaptive weighted fusion dynamically balances branch contributions to boost small target sensitivity. Third, a multi-scale feature fusion mechanism combines upsampling, cross-layer concatenation, and C2FGhostLight-processed features to preserve small target details while maintaining computational efficiency. The backbone’s semantic features are fused with shallow high-resolution features, and the detection head adopts YOLOv8’s optimized Detect structure to enhance small target recognition accuracy. The GS-YOLO architecture is illustrated in Fig 3.

## 4. Research methods

### 4.1. Dataset description

To verify the practical effectiveness of the proposed method in UAV image detection tasks, a series of comparative experiments were designed and conducted, with the VisDrone and UAVDT datasets selected as evaluation benchmarks.

The VisDrone dataset is a large-scale benchmark in UAV vision, widely used for evaluating algorithms in object detection, tracking, and segmentation. Its static image subset covers 10 object categories with a total of 8,629 images, including 6,471 training, 548 validation, and 1,610 test images, providing diverse scenarios for performance assessment and cross-comparison of UAV detection algorithms.

The UAVDT dataset [37] is another commonly used benchmark for UAV aerial image detection. It contains images from 100 video recordings across different cities, scenes, and altitudes, with over 80,000 annotations for cars, trucks, and buses. Due to its large scale, we sampled every 10th frame, resulting in 3,111 images, which were split into training, validation, and test sets at a ratio of 7:2:1 for experimental analysis.

### 4.2. Evaluation metrics

To comprehensively evaluate the detection accuracy and real-time operational efficiency of the model, this paper selects accuracy, recall rate, average precision (AP), average precision mean (mAP), F1 score, and frames per second (FPS) as the core performance measurement indicators. These indicators conduct an assessment of the performance of the detector in the target detection task from multiple dimensions, covering key aspects such as detection accuracy, target recognition completeness, and model inference speed.

1) Precision and Recall: Precision reflects the proportion of actual positive samples among the positive samples predicted by the detector, which is used to characterize the accuracy of the model’s judgment on positive targets; Recall represents the proportion of all actual positive samples that the model successfully identifies, and is mainly used to measure the comprehensiveness of the detector in capturing targets. The specific definitions are as:

$$\begin{array}{c} \text{Precision }=\;\frac{\text{TP}}{\text{TP}+\text{FP}} \end{array}$$ (6)

$$\begin{array}{c} \text{Recall}\;=\;\frac{\text{TP}}{\text{TP}+\text{FN}} \end{array}$$ (7)

2) Average Precision (AP): As the core metric for evaluating the performance of models in the object detection task, AP is used to represent the balance between precision and recall when the model detects specific types of objects. Its value is obtained by calculating the area under the PR curve for that category (AUC), and the formula is defined as follows:

$$\begin{array}{c} \text{AP}=\;\int_{\text{0}}^{\text{1}}\text{P}(\text{r})\text{dr} \end{array}$$ (8)

3) Mean Average Precision (mAP): It represents the average precision of all categories in the dataset, used to evaluate the overall detection performance of the detector across the entire dataset. It is worth noting that m represents the mAP value calculated when the intersection-over-union (IoU) threshold between the predicted box and the real box is set to 0.5. The formula for calculating mAP is as follows:

$$\begin{array}{c} \text{mAP}=\;\frac{\text{1}}{\text{N}}\sum_{\text{i}=\text{1}}^{\text{n}}{\text{AP}}_{\text{i}} \end{array}$$ (9)

4) F1 Score: The F1 score takes into account both precision and recall, and is the harmonic mean of the two. It comprehensively reflects the detection performance of the model. Its calculation formula is as follows:

$$\begin{array}{c} \text{F1 score}=\text{2}\times \;\frac{\text{Precision }\times \text{ Recall}}{\text{Precision }+\text{ Recall}} \end{array}$$ (10)

5) Frame Rate (FPS): As the core metric for measuring the speed of model inference, FPS refers to the number of frames per second (the number of images processed per second), indicating the number of images that the detector can process per second. The higher the FPS value, the stronger the real-time detection performance of the model. The calculation formula is as follows:

$$\begin{array}{c} \text{FPS}=\;\frac{\text{1}}{{\text{T}}_{\text{m}}} \end{array}$$ (11)

### 4.3. Training strategies and implementation details

To ensure the reproducibility of the experimental results, all experiments were conducted on the same high-performance deep learning server. The hardware configuration includes an AMD Ryzen 5 5600 six-core processor and an NVIDIA GeForce RTX 4060Ti-16GB graphics card, with Windows 10 as the operating system. The software environment is built on Python 3.10.18, CUDA 11.8, PyTorch 2.3.1 and TorchVision 0.18.1.

To improve the generalization ability of the model in UAV aerial detection scenarios, we adopt default effective augmentation strategies in the training phase, including Mosaic augmentation, random horizontal flipping, HSV color space adjustment, random erasing and automatic randaugment. These strategies enrich the sample diversity without introducing additional manual operations.

All training hyperparameters are configured strictly according to the experimental settings. For better readability and experimental repeatability, the main training hyperparameters are summarized in Table 1.

**Table 1:** Training hyperparameter settings.

Hyperparameter	Setting
Input image size	640 × 640
Initial learning rate	1 × 10^−2^
Learning rate strategy	Cosine annealing decay
Final learning rate ratio	0.01
Optimizer	SGD
Momentum	0.937
Training epochs	200
Weight decay	5 × 10^−4^
Batch size	8

## 5. Experiments and analysis

### 5.1. Results on VisDrone and UAVDT

**Fig 4:**
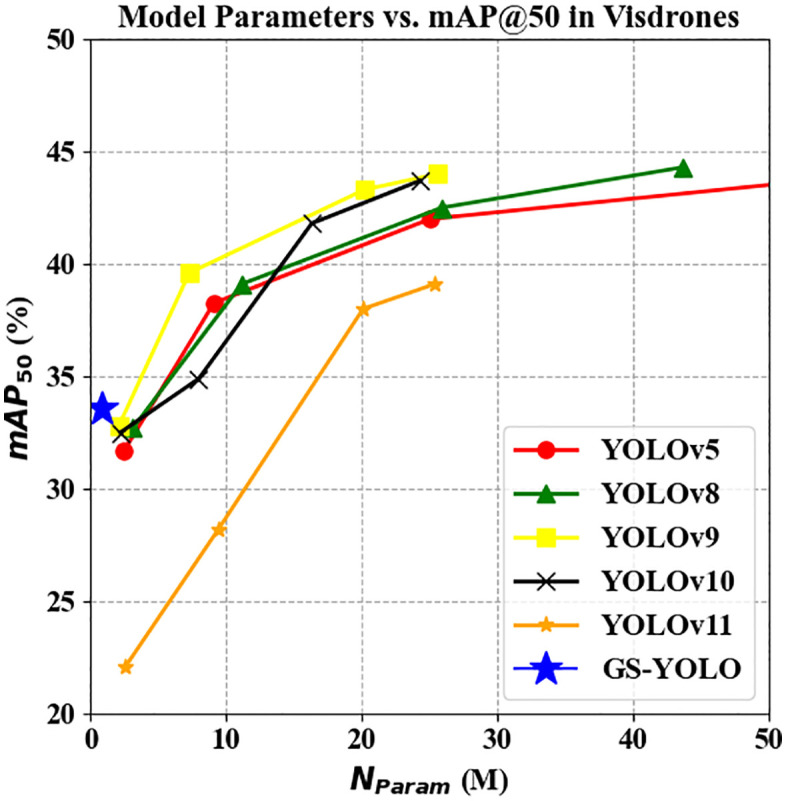
Performance comparison of GS-YOLO and the YOLOv5, YOLOv8, YOLOv9, YOLOv10 and YOLOv11 series on the VisDrone dataset.

Comparison of GS-YOLO with YOLOv5, YOLOv8, YOLOv9, YOLOv10, and YOLOv11 series: To evaluate GS-YOLO’s performance in UAV small target detection, quantitative analysis was performed on the VisDrone test set, with results summarized in Tables 2–6. GS-YOLO was compared against state-of-the-art models from the YOLOv5 to YOLOv11 series, showcasing superior performance in balancing lightweight design, detection accuracy, and real-time capabilities. Fig 4 visually demonstrates GS-YOLO’s superior parameter-accuracy trade-off compared to existing YOLO variants, particularly highlighting its lightweight architecture while preserving detection effectiveness.

**Table 2:** Performance comparison between GS-YOLO and YOLOv5 series.

Method	Params↓	FPS↑	mAP_50_↑	F1↑	P↑	R↑
YOLOv5n	2.51	151	31.7	0.36	0.415	0.32
YOLOv5s	9.12	108	38.26	0.422	0.492	0.371
YOLOv5m	25.07	66	42	0.46	0.553	0.395
YOLOv5l	53.1	49	43.7	0.481	0.548	0.426
GS-YOLO	0.84	70.42	33.6	0.376	0.435	0.331

**Table 3:** Performance comparison between GS-YOLO and YOLOv8 series.

Method	Params↓	FPS↑	mAP_50_↑	F1↑	P↑	R↑
YOLOv8n	3.15	142	32.7	0.3705	0.424	0.328
YOLOv8s	11.16	104	39.1	0.433	0.502	0.38
YOLOv8m	25.90	61	42.5	0.466	0.535	0.414
YOLOv8l	43.69	44	44.3	0.479	0.549	0.425
GS-YOLO	0.84	70.42	33.6	0.376	0.435	0.331

**Table 4:** Performance comparison between GS-YOLO and YOLOv9 series.

Method	Params↓	FPS↑	mAP_50_↑	F1 scores↑	P↑	R↑
YOLOv9t	2.12	99	32.8	0.376	0.44	0.329
YOLOv9s	7.31	84	39.6	0.43	0.5	0.388
YOLOv9m	20.21	48	43.3	0.479	0.557	0.414
YOLOv9c	25.59	45	44.0	0.49	0.56	0.419
GS-YOLO	0.84	70.42	33.6	0.376	0.435	0.331

**Table 5:** Performance comparison between GS-YOLO and YOLOv10 series.

Method	Params↓	FPS↑	mAP_50_↑	F1 scores↑	P↑	R↑
YOLOv10n	2.22	227	32.5	0.373	0.43	0.32
YOLOv10s	8.01	180	34.9	0.389	0.45	0.344
YOLOv10m	16.33	109	41.8	0.454	0.512	0.408
YOLOv10l	24.31	72	43.7	0.475	0.545	0.422
GS-YOLO	0.84	70.42	33.6	0.376	0.435	0.331

**Table 6:** Performance comparison between GS-YOLO and YOLOv11 series.

Method	Params↓	FPS↑	mAP_50_↑	F1 scores↑	P↑	R↑
YOLOv11n	2.61	258	22.1	0.27	0.331	0.269
YOLOv11s	9.45	89.3	28.2	0.329	0.386	0.282
YOLOv11m	20.11	89.9	38	0.428	0.504	0.372
YOLOv11l	25.37	156.25	39.1	0.44	0.528	0.379
GS-YOLO	0.84	70.42	33.6	0.376	0.435	0.331

On the VisDrone dataset, GS-YOLO achieves a 33.6% mAP50 with only 0.84M parameters and a real-time inference speed of 70.42 FPS. Compared to the baseline YOLOv8n, which uses 3.15M parameters and achieves 32.7% mAP50, GS-YOLO reduces parameters by 73.3%, retaining just over a quarter of YOLOv8n’s parameters while improving mAP50 by 0.9%. This demonstrates that its lightweight design effectively minimizes redundancy without sacrificing detection performance. Larger YOLOv8 variants achieve higher mAP50 values but require significantly more parameters and suffer reduced FPS, making them unsuitable for resource-limited UAV platforms.

Compared with YOLOv5 series models, GS-YOLO’s lightweight advantage is further emphasized. YOLOv5n requires 2.51M parameters and achieves only 31.7% mAP50, 1.9% below GS-YOLO. YOLOv5l reaches 43.7% mAP50 but demands 53.1M parameters and 49 FPS, limiting its utility for real-time UAV detection. GS-YOLO, in contrast, balances minimal parameter usage with high accuracy, outperforming competing models in this trade-off.

In comparison to the YOLOv9 series, GS-YOLO excels in lightweight deployment. YOLOv9t has 2.12M parameters and achieves 32.8% mAP50 with 99 FPS, marginally faster than GS-YOLO but at a 2.5 × parameter increase. Larger YOLOv9 models require 7.31M, 20.21M, and 25.59M parameters, with FPS dropping to 84, 48, and 45 — failing to meet UAV resource constraints.

Versus the YOLOv10 series, GS-YOLO strikes a superior lightweight-accuracy balance. YOLOv10n uses 2.22M parameters with a higher 227 FPS but lower 32.5% mAP50. Larger YOLOv10 variants achieve 41.8% and 43.7% mAP50 but require 16.33M and 24.31M parameters, with FPS of 109 and 72. GS-YOLO’s design fills the gap between low-parameter/low-accuracy and high-parameter/high-accuracy models, aligning better with UAV deployment needs.

When compared to the YOLOv11 series, GS-YOLO achieves superior overall performance. YOLOv11n has 2.61M parameters but only 22.1% mAP50, insufficient for UAV small target detection. Larger YOLOv11 variants require 9.45M and 20.11M parameters yet deliver only 28.2% and 38.0% mAP50. Even YOLOv11l, with 25.37M parameters and 156.25 FPS, overcompensates with excessive resource use or speed. GS-YOLO’s 0.84M parameters, 33.6% mAP50, and 70.42 FPS are optimally aligned with UAV deployment requirements.

Comparison with the latest technologies: To better showcase the outstanding performance of GS-YOLO, we conducted a comprehensive comparison of GS-YOLO with other advanced methods on the VisDrone and UAVDT benchmark datasets, including YOLOv5n, YOLOv6n, YOLOv8n, YOLOv9t, YOLOv10n, YOLOv11n, YOLOv12n, YOLOv13n, Hyper-YOLO [38] and Le-YOLO [39]. The experimental results are shown in Table 7.

**Table 7:** Performance Comparison of each model on VisDrone and UAVDT datasets.

Methods	VisDrone						UAVDT					
	Params↓	FPS↑	F1↑	mAP_50_↑	P↑	R↑	Params↓	FPS↑	F1↑	mAP_50_↑	P↑	R↑
YOLOv5n	2.51	151	0.36	31.7	0.415	0.32	2.50	178.57	0.705	73.5	0.815	0.622
YOLOv6n	4.2	126	0.33	29.5	0.399	0.3	4.23	185.19	0.699	72.4	0.787	0.628
YOLOv8n	3.15	142	0.3705	32.7	0.424	0.328	3.0	344.83	0.754	79.0	0.845	0.68
YOLOv9t	2.12	99	0.376	32.8	0.44	0.329	1.97	151.52	0.771	80.9	0.844	0.71
YOLOv10n	2.22	227	0.373	32.5	0.43	0.32	2.27	280	0.689	70.2	0.763	0.629
YOLOv11n	2.61	258	0.27	22.1	0.331	0.233	2.58	222.27	0.703	73.4	0.804	0.624
YOLOv12n	2.5	178	0.366	32.1	0.414	0.328	2.56	256.41	0.680	70.9	0.774	0.606
YOLOv13n	2.44	83.33	0.361	31.4	0.413	0.321	2.45	227.27	0.642	66.4	0.747	0.564
Hyper-YOLO	3.94	133.33	0.394	35.0	0.456	0.346	3.94	121.95	0.772	81.0	0.84	0.715
Le-YOLO	1.89	121.95	0.344	29.2	0.396	0.303	1.89	116.28	0.711	73.5	0.822	0.626
GS-YOLO	0.84	70.42	0.376	33.6	0.435	0.331	0.84	180	0.760	78.5	0.83	0.70

In terms of detection accuracy, GS-YOLO maintains competitive performance across datasets while adhering to an ultra-lightweight design. On VisDrone, it achieves an mAP50 of 33.6—only 1.4% lower than the top-performing Hyper-YOLO—while using just 0.84M parameters compared to Hyper-YOLO’s 3.94M. Its F1-score matches YOLOv9t’s performance and outperforms YOLOv13n and YOLOv5n. On UAVDT, GS-YOLO attains an mAP50 of 78.5, ranking among the top models behind Hyper-YOLO, YOLOv9t, and YOLOv8n, while using less than 28% of the parameters of these top models and significantly outperforming lightweight baselines like YOLOv10n and YOLOv12n.

For lightweight design and real-time performance, GS-YOLO’s core advantage is its minimal parameter count of 0.84M— the smallest among all compared models. This represents dramatic reductions: it uses 33.5% of YOLOv5n’s parameters, 34.4% of YOLOv13n’s, and 21.3% of Hyper-YOLO’s. In terms of speed, it achieves 70.42 FPS on VisDrone and 180 FPS on UAVDT—sufficient for real-time UAV applications and notably faster than larger models such as Hyper-YOLO and Le-YOLO.

Overall, GS-YOLO strikes a superior balance among ultra-lightweight design, cross-dataset accuracy, and real-time performance. With its minimal parameter footprint, it achieves accuracy competitive with much larger models while delivering frame rates suitable for real-time UAV deployment.

### 5.2. Analysis of AP performance by categories

To further analyze the performance differences of the model in detecting different types of targets, this section conducts a statistical and comparative analysis of category AP values on the VisDrone and UAVDT datasets. The quantitative results are presented in Table 8. The proposed GS-YOLO shows obvious performance differentiation across target categories. Overall, it achieves superior detection accuracy for conventional vehicle targets, whereas its detection capability for pedestrians and non-motorized vehicles is substantially degraded.

**Table 8:** Analysis of AP Performance in Visdrone and UAVDT Categories.

Methods	Visdrones	UAVDT
	mAP_50_	mAP_50_
Pedestrian	0.394	\
People	0.32	\
Bicycle	0.0784	\
Car	0.781	0.801
Van	0.389	0.381
Truck	0.248	0.518
Tricycle	0.202	\
Awning-tricycle	0.116	\
Bus	0.441	0.754
Motor	0.389	\

On the VisDrone dataset, the model presents evident performance differences across vehicle categories. The Car achieves the highest AP of 0.781, benefited from its regular shape, stable distribution and prominent structural features in UAV aerial imagery, which support effective feature learning. Bus, Van and Motor attain steady and moderate accuracy, demonstrating the model’s competitive capability in detecting conventional motor vehicles.

For pedestrian detection, performance varies slightly across subclasses. Pedestrian obtains an AP of 0.394, while People reaches 0.320. The slight drop is mainly caused by dense crowd distribution, mutual occlusion, small target pixels and blurred outlines, which increase the difficulty of feature representation in complex scenarios.

In terms of non-motorized vehicles, Bicycle, Tricycle and Awning-tricycle obtain relatively lower AP values due to their small target scale, irregular structure, inconspicuous texture, and easy confusion with complex background and occluded interference in aerial scenes, which brings certain challenges to feature discrimination. Future work will focus on optimizing the detection of small-sized non-motorized vehicles and densely occluded pedestrians. Multi-scale feature enhancement and occlusion-aware feature fusion will be adopted to strengthen the model’s feature extraction for challenging samples, so as to improve its robustness and generalization in complex UAV aerial scenarios.

On the UAVDT dataset, GS-YOLO maintains favorable and stable accuracy on major vehicle classes, showing promising cross-dataset generalization and application feasibility for practical UAV detection tasks.

### 5.3. Ablation analysis

To verify the effectiveness of each improvement strategy in the GS-YOLO, this study conducted ablation experiments, the corresponding results are compiled in Table 8. This table illustrates the detection performance achieved on the VisDrone test set after incorporating different improvement components into the YOLOv8n base model. Experimental findings indicate that each improvement component exerts a positive enhancement effect on detection precision.

Table 9 presents the ablation experiment results on the VisDrone test set based on the YOLOv8n baseline model, with the introduction of the C2FGhostLight module, the SOLCA module, and their combination. The experimental data shows that each individual component can positively enhance the detection performance: when only the C2FGhostLight module is added, the model parameter size decreases from 3.15M to 0.83M, with a significant lightweight effect, and mAP50 increases to 32.9%; when only the SOLCA module is added, the parameter size is controlled at 0.86M, and mAP50 increases to 33%, with slight improvements in precision (P) and recall (R). When the two components work together, the model exhibits a “1 + 1>2” synergistic gain: the parameter size remains at the extreme lightweight level of 0.84M, mAP50 further increases to 33.6%, compared to the baseline, it increases by 0.9%, and both precision (0.435) and recall (0.331) reach the optimal level. Although FPS drops to 70.42, it still meets the real-time deployment requirements, fully verifying the synergy effectiveness of the C2FGhostLight and SOLCA modules in lightweight feature extraction, background suppression, and small target perception. The combination of the two achieves the optimal balance between lightweight and detection accuracy.

**Table 9:** Performance Comparison of baseline combinations under Different Strategies.

Method	C2FGhostLight	SOLCA	Params	mAP_50_	FPS	P	R
Baseline	-	-	3.15	32.7	142	0.424	0.328
	√	-	0.83	32.9	120.48	0.426	0.327
	-	√	0.86	33	90.9	0.43	0.33
	√	√	0.84	33.6	70.42	0.435	0.331

To verify the rationality and optimal value of the residual attenuation coefficient in formula (3) of 3.1, a set of gradient ablation experiments was designed: The residual attenuation coefficient β was set to 0.0, 0.2, 0.4, 0.6, 0.8, 1.0, and 1.2 respectively. Under the condition that the other structures of the model remained unchanged, only the value of β was adjusted. The core performance indicators of the model were tested on the VisDrone dataset to determine the optimal coefficient that balances detection accuracy and inference efficiency. The experimental results are shown in Table 10.

**Table 10:** Performance comparison of different residual attenuation coefficients β on the VisDrone dataset.

Residual attenuation coefficient β	FPS	mAP_50_
0.0	72.5	30.1
0.2	71.9	31.8
0.4	71.2	32.7
0.6	70.8	33.2
0.8	70.42	33.6
1.0	70.1	33.0
1.2	69.8	32.5

The experimental results based on the residual attenuation coefficient β show that as β gradually increases from 0.0 to 0.8, the mAP50 of the model on the VisDrone dataset continuously improves and reaches the optimal value of 33.6% at β = 0.8. The FPS decreases steadily from 72.5 to 70.42. While the accuracy significantly improves, the inference efficiency remains stable. When β exceeds 0.8, the mAP50 of the model significantly decreases, and the FPS further drops. This is because excessive residual weights will mask the multi-branch enhanced features and disrupt the balance of feature fusion. The comprehensive performance comparison indicates that β = 0.8 can achieve the optimal balance between avoiding the absence of basic features and preventing the enhanced features from being masked. It is a reasonable value suitable for the small target detection scenario of unmanned aerial vehicles and the lightweight architecture of GS-YOLO. Its selection has sufficient experimental basis.

### 5.4. Visual analysis

**Fig 5:**
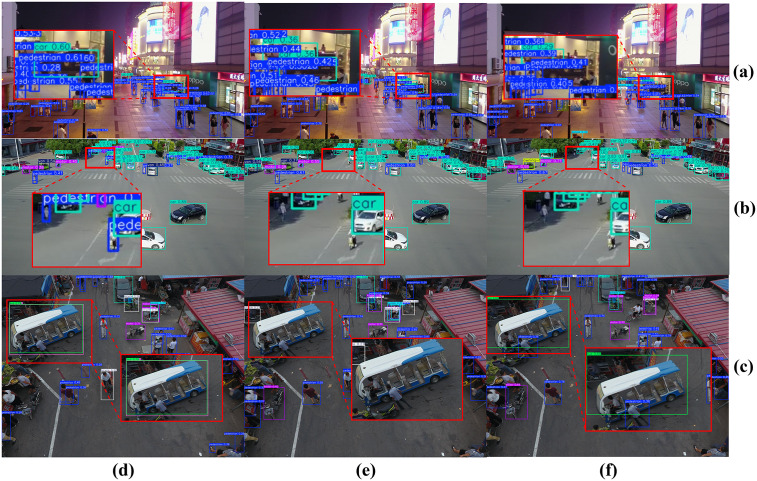
Comparison of Visualization of GS-YOLO and YOLO Series. Rows **(a)**, **(b)** and **(c)** show different scenarios, while columns **(d)**, **(e)** and **(f)** present the detection results of GS-YOLO, YOLOv12n and YOLOv13n, respectively. (The visualization images in this figure are generated based on the original images from the public VisDrone dataset used in this study. These images include algorithm-generated detection bounding boxes, confidence score labels, and manually annotated red boxes and dashed lines that highlight representative detection comparison regions. The original VisDrone dataset images are raw, unannotated aerial photographs, so the presented visualization images are similar in scene content but not identical to the original dataset images, and are therefore for illustrative purposes only.).

Fig 5 presents the comparison chart among the models of the Visdrone dataset. In the night street scenario (a), GS-YOLO achieves accurate detection and clear annotation for all objects, including distant small targets at image edges, with almost no missed detections or false positives, while YOLOv12n suffers from partial small-object omissions and inaccurate localization, and YOLOv13n exhibits more severe missed detections. For the aerial-view urban intersection scenario (b) with complex backgrounds, GS-YOLO demonstrates excellent full-scale detection capability, accurately annotating vehicles and pedestrians with tight, well-aligned bounding boxes and no small-object misses in zoomed areas; in contrast, YOLOv12n shows obvious small-object omissions and localization offsets, and YOLOv13n further suffers from expanded missed detections and severe box misalignment. In the dense market scenario (c), GS-YOLO accurately identifies large buses and captures surrounding small pedestrians and facilities with high annotation precision, while YOLOv12n has reduced small-object accuracy and increased misses, and YOLOv13n shows significant omissions and insufficient box coverage. Overall, GS-YOLO outperforms the two baselines in all complex environments, effectively addressing the detection challenges of small and dense objects, improving localization accuracy, and ensuring robust category labeling, making it more suitable for challenging real-world visual detection tasks.

**Fig 6:**
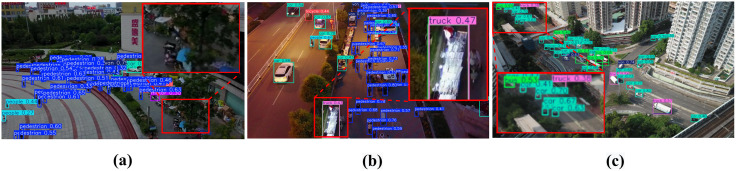
Detection results of GS-YOLO on the VisDrone dataset: Occluded and high-altitude extremely small target scenarios.

In the actual measurement of the VisDrone dataset, GS-YOLO demonstrated a differentiated performance characterized by “stable recognition of very small targets, but limited performance in extreme occlusion scenarios”. This characteristic is clearly illustrated in Fig 6. The model shows strong robustness in recognizing very small targets from the high-altitude perspective of the drone, but has shortcomings in identifying the occluded targets in dense occlusion scenarios.

In scene (a), the densely arranged pedestrians caused a large number of detection failures due to occlusion. Additionally, the trees in the lower right corner partially blocked the vehicles, resulting in some vehicles obscured by the trees being completely missed. In scene (b), some partially occluded pedestrians lost their key local features, thus being misclassified as motor vehicles. The occluded target branches experienced a decrease in cosine similarity with the global features due to the loss of features. In the urban intersection scene (c), GS-YOLO demonstrated outstanding ability in detecting very small vehicles at high altitudes. It successfully identified numerous small vehicles on different lanes without large-scale omissions and achieved precise positioning. The continuous challenges faced by GS-YOLO in extreme occlusion scenarios highlight the limitations of the current feature fusion mechanism in dealing with the absence of local features. This difference clearly indicates the optimization direction that future models based on drones should take when improving in complex environments.

## 6. Conclusion

Aiming at the core challenges of weak feature representation, severe background interference, and the lightweight-deployment versus detection-accuracy trade-off in UAV small target detection, this paper proposes the GS-YOLO lightweight high-precision detection framework. The framework incorporates the C2FGhostLight lightweight attention fusion module and the SOLCA small-target perception module, integrating multi-scale feature fusion to enable end-to-end lightweight detection. This design effectively addresses key limitations of UAV small-target detection. Experimental results on VisDrone and UAVDT datasets confirm the model’s superiority: on VisDrone, it achieves 33.6% mAP50 and 70.42 FPS real-time inference with just 0.84M parameters, outperforming YOLOv8n and mainstream lightweight YOLO models. On UAVDT, it attains 78.5% mAP50 with strong performance in high-altitude ultra-small target detection. Ablation studies further validate the synergistic enhancement of the core modules and the rationality of residual attenuation coefficient settings.

GS-YOLO has an obvious practical limitation in extreme occlusion scenarios: visual analysis shows significant detection failures for densely overlapping pedestrians and targets occluded by trees, stalls or non-motor vehicles. For this limitation, future research will focus on optimizing the model’s feature fusion and weight allocation mechanisms for occlusion scenarios on the premise of retaining its ultra-lightweight design to enhance the feature extraction capability of occluded small targets, and simultaneously improve the model’s deployment efficiency through lightweight technologies such as quantization.

In summary, GS-YOLO achieves a robust balance between ultra-lightweight design, real-time inference, and detection accuracy in UAV small-target scenarios, offering a viable solution for lightweight UAV detection systems. Enhancements for occlusion robustness will further strengthen its performance in complex environments, positioning it as a valuable tool for UAV surveillance, precision agriculture, and infrastructure inspection.
